# Assessment of enzymatic browning in blanched potato slices using combined hyperspectral imaging and hyperspectral microscopy imaging

**DOI:** 10.1016/j.fochx.2025.103356

**Published:** 2025-11-30

**Authors:** Jong-Jin Park, Sang Seop Kim, Seul-Ki Park, Gyuseok Lee, Kee-Jai Park, Jeong-Ho Lim, Jeong-Hee Choi, Jeong-Seok Cho

**Affiliations:** a Smart Food Manufacturing Research Group, Korea Food Research Institute, Wanju-gun 55365, South Korea; b Food Convergence Research Division, Korea Food Research Institute, Wanju-gun 55365, South Korea; c Department of Food Biotechnology, University of Science and Technology, Daejeon 34113, South Korea

**Keywords:** Potato, Browning, Hyperspectral imaging, Hyperspectral microscope imaging, Polyphenol oxidase, Phenolic compounds

## Abstract

Inadequate blanching in fresh-cut potatoes causes browning during storage, but it cannot be detected immediately post-processing. Moreover, browning patterns during storage vary with blanching conditions. Therefore, predictive models based on hyperspectral imaging (HSI) and hyperspectral microscopy imaging (HMI) were developed to investigate blanching-dependent alterations in polyphenol oxidase (PPO) activity and total phenolic content (R_p_^2^ = 0.71–0.87). HSI visualization maps showed a spatial concordance between regions exhibiting high residual PPO and storage-induced browning. HMI maps revealed mild and extensive browning in untreated samples due to an intact cellular structure delaying PPO-phenolic compound interactions, and strong but localized browning in insufficiently blanched samples due to partial cell disruption, which facilitated enzyme-substrate interactions. Contrastingly, sufficiently blanched samples did not exhibit browning due to PPO inactivation. These results suggest that HSI and HMI integration can function as a monitoring tool for early detection of browning and improve our understanding of the underlying mechanisms.

## Highlights

• We used hyperspectral imaging (HSI) + −microscopy (HMI) to analyse potato browning.
• HSI + HMI, combined with PLSR models, can predict PPO activity and TPC.
• Spatial–spectral information from HSI and HMI offers insights into enzymatic browning phenomena.

## 1 Introduction

Potatoes (*Solanum tuberosum* L.) are among the most widely consumed crops worldwide because of their nutritional value, versatility, and affordability. The growing demand for fresh-cut, ready-to-cook vegetables has accelerated the development of minimally processed potato products (Levaj et al., 2023). However, enzymatic browning remains a major obstacle, as it reduces visual quality, consumer acceptance, and commercial value (Hou et al., 2014).

Enzymatic browning is primarily caused by the oxidation of phenolic compounds catalysed by polyphenol oxidase (PPO). PPO converts phenolic substrates into quinones, which polymerise into dark pigments (Tsikrika et al., 2022). Accordingly, PPO activity and phenolic content have been widely monitored as browning indicators in food products (Wang et al., 2020).

However, fresh-cut potatoes display substantial variation in browning location, intensity, and rate depending on processing conditions and sample types. Nevertheless, conventional quantitative assays of browning indicators alone are limited in their ability to fully explain this variation. Accordingly, some studies have attempted to analyse the browning phenomenon from multiple perspectives using diverse techniques. Wang et al. (2020) compared PPO activity with PPO gene expression to better understand cultivar-dependent browning patterns following wounding. Oey et al. (2017) employed various microscopic techniques (light, fluorescence, and scanning electron microscopy) and the tetrazolium cell viability staining assay to interpret the differences in browning area and intensity under different pulsed electric field treatments. Collectively, previous studies highlighted that PPO activity, phenolic compounds and the state of cell compartmentalization are key determinants of browning behaviour in potatoes. However, conventional assays for measuring PPO activity and phenolic compounds are destructive, labour-intensive, and time-consuming, whereas the assessment of cell compartmentalization through microscopy provides only structural information and is limited in delivering chemical compositional information unless reagent pretreatment is performed.

In this study, hyperspectral imaging (HSI) was employed as a promising approach to address these limitations. HSI, which integrates computer vision and spectroscopy, is a non-destructive, non-invasive, chemical-free, and rapid technique that enables accurate chemical and spatial analyses (Siripatrawan & Makino, 2025). Previous studies have demonstrated its potential for quantifying the PPO activity and phenolic content in various crops, including potatoes and fruits (Ciccoritti et al., 2025; Shrestha et al., 2020).

In addition, this study employed hyperspectral microscopy imaging (HMI), which combines HSI with microscopy to provide micron-level spatial resolution (Pu et al., 2019). HMI has been applied in materials science, environmental monitoring, and biomedicine (Banu et al., 2024). Recent studies have demonstrated its utility in detecting anthocyanins in potatoes, chlorophyll in matcha, and the microstructural features of blueberries (Li et al., 2024; Park et al., 2023; Wang et al., 2024). Nevertheless, the broader potential of HMI for food quality assessment remains underexplored.

This study focused on blanching conditions. Blanching is a widely applied thermal pretreatment to inactivate browning-related enzymes and reduce microbial load. However, insufficient blanching can accelerate browning during distribution and storage, which may raise concerns regarding consumer safety, reduce product marketability, and lead to economic losses. To our knowledge, no studies have simultaneously employed HSI and HMI to monitor browning indicators in blanched potatoes under different conditions. Considering the distinct capabilities of each technique, HSI is expected to visualize browning-related compounds in different tissues (e.g., perimedullary zone, vascular ring, and cortex), thereby revealing the browning patterns of fresh-cut potatoes, whereas HMI is anticipated to enable simultaneous observation of cellular structure and compositional attributes at the microscopic scale (Banu et al., 2024). Based on this complementarity, we hypothesized that integrating HSI and HMI would allow a more comprehensive understanding of the browning phenomenon.

Consequently, this study aimed to obtain multiscale spatial information on browning indicators (PPO and TPC) of fresh-cut potatoes using hyperspectral imaging data and to understand the differences in browning patterns under different blanching conditions based on these results.

## 2 Materials and methods

### 2.1 Materials

Potatoes harvested in 2024 from Dangjin, Chungcheongnam-do, were washed, peeled, and cut into cylindrical slices with a height of 1 cm and a diameter of approximately 7 ± 0.5 cm.

### 2.2 Blanching procedure and measurement of internal temperature

The potato slices were blanched at 80 °C in a water bath (BS-21, Lab Companion, Korea) for 0, 2, 4, 6, or 10 min in a **sample-to-distilled-water ratio of 1:10 (*w*/*v*),** followed by immediate cooling in cold water for 1 min. After cooling, the samples were drained and gently wiped with paper towel, **and then used for subsequent experiments**. The internal temperature of three randomly selected potato slices was measured immediately after blanching by inserting a probe into their geometric centres, and the temperature was recorded using a digital food thermometer (FT-500, Zhongshan Hyperda, China).

### 2.3 Polyphenol oxidase (PPO) activity

PPO activity was measured using a commercial assay kit (Qarigo Biolaboratories, ARG82015) following the manufacturer's protocol. Potato tissue (0.3 g) was frozen with liquid nitrogen, ground, and homogenised with 1 mL assay buffer, then centrifuged at 13,000 rpm for 10 min at 4 °C using a Smart-R17 centrifuge (Hanil Science Industrial, Incheon, Korea). The supernatant (50 μL) was mixed with 150 μL of reaction buffer and 50 μL of substrate, and the mixture was incubated at 37 °C for 3 min. After adding stop solution (100 μL), the sample was centrifuged at 13,000 rpm for 10 min. The absorbance of supernatant was measured at 410 nm using a microplate reader (Infinite M200 PRO, Tecan, Mannedorf, Switzerland). The results were expressed as units per gram of fresh weight.

### 2.4 Total polyphenol content (TPC)

The total polyphenol content was measured using the Folin–Ciocalteu method, with slight modifications (Singleton, 1999). A lyophilised sample (0.5 g) of potatoes was mixed with 10 mL of 80 % ethanol and extracted for 30 min using the Powersonic 510 ultrasonic machine (Hwashin Technology, Seoul, Korea). After extraction, the sample was centrifuged at 4000 rpm for 20 min. The supernatant (200 μL) was mixed with 1 N of Folin–Ciocalteu's reagent (200 μL) and 5 % Na_2_CO_3_ (750 μL). The mixture was allowed to react for 20 min at room temperature. Absorbance was measured at 760 nm using the microplate reader. Gallic acid was used as the standard, and the results were expressed as milligrams per 100 g.

### 2.5 RGB images and colour values during storage

The samples were maintained at a temperature of 25 °C for 6 h and 24 h. Throughout this period, RGB images were captured inside an illumination chamber measuring 110 × 110 × 150 cm, which was designed to block out external light. The chamber was fitted with light-emitting diodes (LEDs; TSM Co., Ltd., Gwangmyeong-si, Korea) and a digital camera (AT-200GE; Jai Ltd., Kanagawa, Japan). The regions of interest were identified using Python software (version 3.11; Python Software Foundation, Wilmington, USA). Subsequently, a colour board (SpyderCheckr, Datacolor Inc., NJ, USA) was used for calibration, and the lightness (L*), red/green axis (a*), blue/yellow axis (b*), and colour difference (△E) values of the samples were determined. The △E value is calculated as follows (Mokrzycki & Tatol, 2011):

$$∆E=\sqrt{{\left(L^{*}-{L^{*}}_{0}\right)}^{2}+{\left(a^{*}-a_{0}^{*}\right)}^{2}+{\left(b^{*}-b_{0}^{*}\right)}^{2}}$$

### 2.6 Hyperspectral imaging

Hypercubes of the sliced potatoes were collected using an HSI system that included a pushbroom line-scan specimen scanner system (FX10e, Spectral Imaging Oy Ltd., Oulu, Finland), with the module moving steadily at a speed of 10 mm/s. Data were obtained within the VIS/NIR spectral range of 600–1000 nm. The region of interest was determined using the principal component analysis (PCA)-based ROI feature in the Prediktera Breeze software (Prediktera AB, Umea, Sweden), and the average spectrum for each sample was extracted for subsequent analysis.

### 2.7 Hyperspectral microscope imaging

To expose the cross-sectional surface, potato slices were horizontally sectioned, and samples were selected from the perimedullary zone and vascular ring regions that exhibited distinct browning patterns during storage experiments depending on blanching treatment. After cutting the potatoes into 20 × 20 × 0.2 mm pieces using a razor blade, they were placed on quartz glass slides for HMI analysis. The hyperspectral imaging microscope was a custom-made system comprising a microscope (CX41, Olympus, Japan), visible acousto-optic tuneable filter (AOTF) module (VA410–400-1000-ER, Bimrose, USA), and a camera (ASI678, ZWO, China). The AOTF module is an optical device that selects and outputs some of the wavelengths of incidence light, with specifications of a wavelength range of 600–1000 nm, a spectral resolution of 3–8 nm, and an active aperture of 10 × 10 mm. The camera was selected based on its compatibility with the active aperture of the AOTF module, featuring specifications such as a sensor (SONY IMX678), sensor format (1/1.8″), sensor size (7.68 × 4.32 mm), resolution (3840 × 2160), maximum frame rate (47.5 fps), and exposure range (32 μs–2000 s). The AOTF module was added at the pre-camera connection stage in a conventional microscope system capable of general imaging, enabling the sequential acquisition of images at selected wavelengths by operating the AOTF module. The 10× magnification was used for HMI analysis. For accurate and stable HMI, the collected original image was calibrated to eliminate the dark current effect and reduce the influence of uneven illumination distribution.

### 2.8 Partial least squares regression (PLSR) model and visualising the distribution map

PLSR analysis was performed using the Python software. The data were divided into two subsets: 75 % served as the training set, and the remaining 25 % was used for the prediction model. A random 10-fold cross-validation method was employed to further split the training data into calibration and validation subsets. The effectiveness of the model was evaluated by the coefficients of determination for the calibration (R_c_^2^), cross-validation (R_cv_^2^), and prediction (R_p_^2^) datasets. The assessment metrics included the root mean square errors for calibration (RMSEC), cross-validation (RMSECV), and prediction (RMSEP). The ratio of prediction deviation (RPD) was determined by dividing the standard deviation of the reference data from the calibration set by the RMSEP (Huang & Lu, 2010). Pixel-wise spectral data were processed using the established PLSR model, and the distribution maps of PPO activity and TPC were visualised using the Python software.

### 2.9 Statistical analysis

Tukey's honestly significant difference (HSD) test was conducted using IBM SPSS Statistics 25 (SPSS Inc., Chicago, IL, USA) with significance set at *p < 0.05*. RGB imaging and colour measurements during the storage experiment were performed in 10 replicates, whereas the hypercube, PPO activity, and TPC results were collected from 15 replicates.

## 3 Results and discussion

### 3.1 Comparison of browning in potatoes during storage under different blanching conditions

**Fig. 1:**
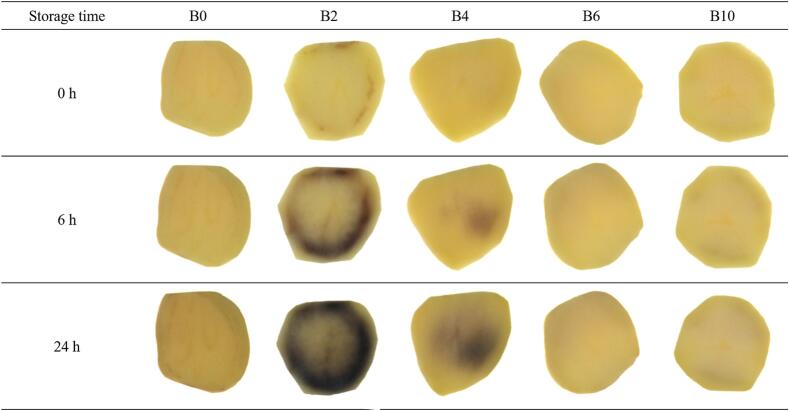
Red-green-blue **(**RGB) images of potato samples by blanching and storage times. (For interpretation of the references to colour in this figure legend, the reader is referred to the web version of this article.)

The RGB images and colour values of the potatoes under different blanching conditions were measured (Fig. 1 and Table 1). Immediately after blanching, the colour difference (ΔE) for all treatments was <5 and ΔE showed no significant difference in terms of blanching time (*p > 0.05*). However, the L* and b* values of fresh-cut potato decreased with increasing storage time, whereas the a* value increased. This change indicates that, in the presence of oxygen, phenolic compounds were oxidized by browning-related enzymes into quinones, which subsequently polymerized into dark-colored polymeric pigments (Sui et al., 2023).

**Table. 1:** Colour values of potato slices by blanching and storage times.

	Storage time (h)	B0	B2	B4	B6	B10
L*	0	59.7 ± 1.5^bc^	56.8 ± 2.2^ab^	56.0 ± 2.3^a^	59.0 ± 3.2^abc^	60.9 ± 2.7^c^
	6	56.4 ± 1.8^bc^	48.4 ± 3.3^a^	54.9 ± 4.4^b^	58.4 ± 2.5^bc^	59.5 ± 2.0^c^
	24	49.9 ± 2.3^b^	33.3 ± 6.3^a^	50.7 ± 4.9^b^	56.2 ± 3.5^c^	57.4 ± 1.7^c^
a*	0	−2.4 ± 1.1^a^	−1.8 ± 1.2^a^	−2.0 ± 1.3^a^	−2.1 ± 1.1^a^	−2.7 ± 1.1^a^
	6	−2.2 ± 1.2^a^	1.0 ± 1.2^b^	−1.3 ± 1.9^a^	−2.0 ± 1.1^a^	−2.3 ± 1.1^a^
	24	0.7 ± 1.3^bc^	1.6 ± 0.8^c^	−0.4 ± 1.7^ab^	−0.6 ± 1.4^ab^	−1.2 ± 1.4^a^
b*	0	36.0 ± 1.1^a^	36.2 ± 1.5^a^	37.0 ± 1.8^a^	37.7 ± 2.0^a^	39.8 ± 1.2^b^
	6	34.8 ± 1.2^b^	28.3 ± 2.9^a^	34.0 ± 4.3^b^	35.7 ± 2.4^b^	37.1 ± 1.2^b^
	24	33.1 ± 1.5^bc^	17.1 ± 5.3^a^	29.1 ± 4.8^b^	31.9 ± 3.2^bc^	33.8 ± 2.1^c^
∆E	0	–	3.6 ± 1.9^b^	4.5 ± 1.9^a^	3.7 ± 2.0^a^	4.7 ± 1.8^a^
	6	3.9 ± 1.8^a^	14.2 ± 4.3^b^	6.4 ± 5.2^a^	3.3 ± 1.9^a^	2.5 ± 1.0^a^
	24	10.8 ± 2.3^b^	32.8 ± 8.0^c^	11.8 ± 6.5^b^	6.1 ± 4.4^ab^	4.0 ± 2.0^a^

Different superscript lowercase letters in same row indicate significant differences (*p < 0.05*).

Meanwhile, the degree of colour change and the pattern varied depending on the blanching condition. After 24 h of storage, B0 samples showed a ∆E of 10.8 with mild browning occurring across the surface. The ∆E values of B6 and B10 samples were 6.1 and 4.0, respectively, which were lower than that of B0 samples, indicating that sufficient blanching (B6 and B10) effectively suppressed browning. In contrast, B2 samples reached a ∆E of 32.8 and exhibited strong browning that initiated near the vascular ring and spread throughout the tissue. The ∆E value of B4 samples (11.8) was not significantly different from that of B0 samples (*p > 0.05*); however, browning in B4 samples was mainly localized. These findings confirm that insufficient blanching (B2 and B4) can accelerate browning during storage. Previous studies reported that the initial levels and changes during storage of browning enzymes and total phenolic content (TPC) vary among different potato tissues, which in turn, leads to tissue-specific differences in the degree of browning over time (Wang et al., 2020). Therefore, in the next step, PPO activity and TPC under each blanching condition were measured to further investigate the relationship between blanching time and browning.

### 3.2 Browning indicator changes in potatoes under different blanching conditions

**Fig. 2:**
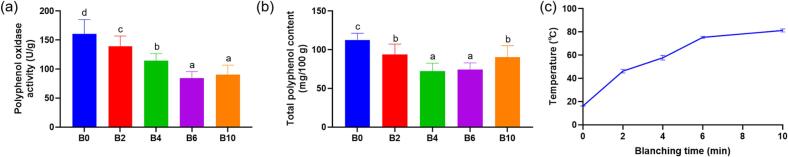
(a) Polyphenol oxidase activity, (b) total polyphenol content, and (c) temperature profiling of potato samples by blanching time.

The PPO activity and TPC of potatoes were measured according to blanching time (Fig. 2a and b). Previous study reported that PPO retained its catalytic activity up to approximately 45 °C, but between 45 °C and 70 °C its activity gradually decreased due to protein denaturation, characterized by decreases in α-helix and β-sheet contents and increases in aggregated β-sheets, turns, and random coil structures (Baltacıoğlu et al., 2015). Above 70 °C, PPO becomes irreversibly inactivated by disruption of the tertiary structure (Baltacıoğlu et al., 2015; Iqbal et al., 2018; Yemenicioğlu, 2002).

As shown in Fig. 2a, PPO activity decreased progressively with increasing blanching time, indicating that thermal exposure effectively reduced enzyme activity. However, despite the treatment temperature being 80 °C, B2 and B4 still exhibited a certain level of PPO activity sufficient to initiate browning during storage (Fig. 1, Fig. 2a). This observation can be attributed to the fact that the potato core did not reach the immersion temperature during the blanching process, thereby limiting enzyme inactivation (Abu-Ghannam & Crowley, 2006). Consistent with this explanation, the internal temperatures of B2 and B4 samples were 46.3 °C and 57.8 °C, respectively, which were insufficient for PPO inactivation (Fig. 2c). In contrast, the internal temperatures of B6 and B10 samples exceeded 75 °C, resulting in a marked decrease in PPO activity (Figs. 2a and c). These results confirm that non-uniform internal temperature distribution during blanching may result in incomplete enzyme inactivation within potato tissues, thereby promoting browning during storage.

As blanching progressed, TPC decreased in B6 samples, whereas in B10 samples it significantly increased to 93.2 mg GAE/100 g (*p < 0.05*) (Fig. 2b). The decrease in TPC in B6 samples was likely due to leaching or thermal degradation of phenolic compounds (Kaiser et al., 2012), whereas the subsequent increase in TPC in B10 samples may have resulted from enhanced extractability of polyphenols following heat-induced tissue disruption (Mowafy & Liu, 2024). According to previous studies, PPO exerts a greater influence on enzymatic browning than TPC (Wang et al., 2020). Therefore, the effective suppression of browning observed in B10 samples, despite the increase in TPC, can be attributed to the substantial reduction in PPO activity.

However, in B2 and B4 samples, tissue-specific browning differences occurred despite significant decreases in both PPO activity and TPC compared with B0 samples, which could not be fully explained by compositional analysis alone. Therefore, it was considered necessary to obtain spatial information on the distribution of browning indicators. To address this, HSI, which can quantitatively estimate and visualize PPO and TPC (Hu et al., 2024; Yang et al., 2015), was applied. Consequently, in the next step, quantitative HSI-based models were developed to estimate these two browning indicators under each blanching condition.

### 3.3 Quantification of PPO activity and TPC using HSI and HMI

**Fig. 3:**
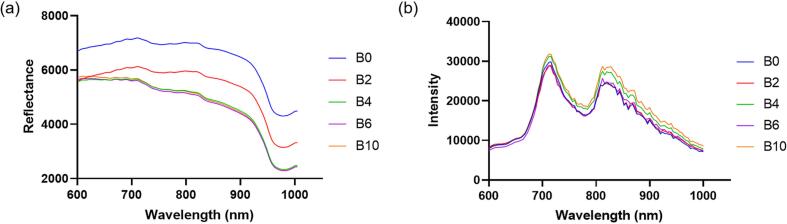
Mean spectrum of potato samples by blanching time using (a) hyperspectral imaging and (b) hyperspectral microscopy imaging.

**Fig. 4:**
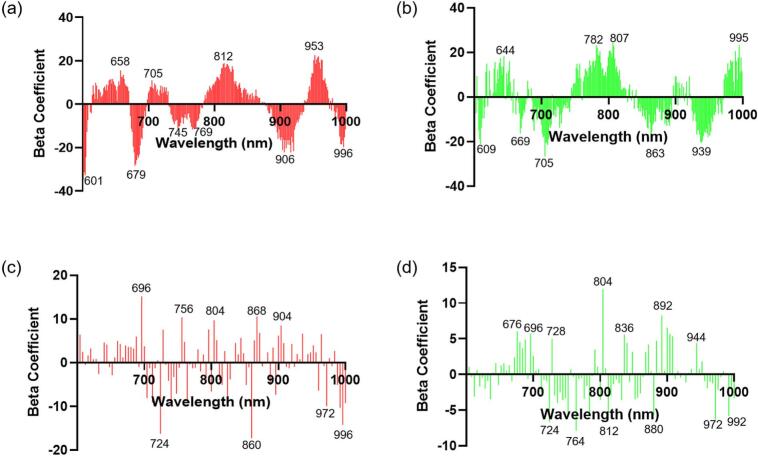
Beta-coefficients of partial least squares regression models for quantifying polyphenol oxidase (PPO) activity and total polyphenol content (TPC). (a) PPO activity and (b) TPC using hyperspectral imaging, and (c) PPO activity and (d) TPC using hyperspectral microscopic imaging.

The spectra of potato slices with different blanching times obtained using HSI are shown in Fig. 3. The mean HSI spectrum exhibited higher reflectance for unblanched samples (B0) than for the blanched samples. Peaks were observed at 705 nm and 800 nm, whereas valleys were observed at 750 and 985 nm. In contrast, these peaks and valleys, which were less distinct in the HSI analysis, appeared more prominently in HMI analysis, with distinct peaks at approximately 716 and 812 nm and a pronounced valley near 784 nm. (See Fig. 4.)

Using the spectral data derived from the HSI and HMI, PLSR models were constructed to predict PPO activity and TPC. The PLSR model results for TPC and PPO activity are presented in Table 2. The PLSR models revealed that HMI achieved superior performance for PPO prediction (R_p_^2^ = 0.87, RPD = 3.03), whereas HSI showed slightly lower but reliable accuracy (R_p_^2^ = 0.82, RPD = 2.24). For PPO activity prediction, the wavelengths that exhibited high beta coefficient values were 601, 658, 679, 705, 745, 769, 812, 906, 953, and 996 nm for HSI, and for HMI, the significant wavelengths were 696, 724, 756, 804, 860, 868, 904, 972, and 996 nm. Wavelengths of 688 nm and 800 nm are closely associated with the O—H and C—H chemical bonds, which are key indicators of PPO (Yang et al., 2015). The superior PPO prediction performance of the HMI-based model may be attributed to the shorter optical path length and reduced scattering within thin microscopic sections, which minimize spectral averaging effects and enhance sensitivity to localized biochemical variations (Quintana-Quintana et al., 2025).

**Table 2:** Results of partial least squares regression for polyphenol oxidase (PPO) activity and total polyphenol content (TPC) quantification using hyperspectral imaging (HSI) and hyperspectral microscope imaging (HMI).

Types	Parameter	Calibration		Cross-validation		Prediction		RPD
		R_c_^2^	RMSEC	R_cv_^2^	RMSECV	R_p_^2^	RMSEP	
HSI	PPO activity	0.91	10.02	0.81	13.24	0.82	14.59	2.24
	TPC	0.84	7.24	0.68	9.39	0.71	10.26	1.76
HMI	PPO activity	0.94	8.16	0.82	13.23	0.87	11.21	3.03
	TPC	0.86	6.65	0.72	9.49	0.72	10.17	1.78

R^2^, coefficient of determination; RMSEC, RMSECV, and RMSEP, root-mean-square error of calibration, cross-validation, and prediction, respectively; RPD, ratio of prediction deviation.

For TPC prediction, both HSI and HMI showed similar results, with R_p_^2^ values of 0.71 and 0.72 and RPD values of 1.76 and 1.78, respectively. These RPD values (1.5 < RPD < 2) indicated that the model could distinguish between low and high values of the response variables (Nicolai et al., 2007). For TPC prediction, the wavelengths with high beta coefficient values were 609, 644, 669, 705, 782, 807, 863, 939, and 995 nm for HSI. In contrast, for HMI, the corresponding wavelengths were 676, 696, 724, 728, 764, 804, 812, 836, 880, 892, 944, 972, and 992 nm. Absorption in the 730–770 nm range is attributed to the tertiary frequency of C—H bonds, which provides compositional information on phenolic compounds (Ji et al., 2019). Additionally, the absorption observed in the 657–839 nm range may correspond to the third overtone of N—H stretching, which is typically linked to the protein content in the samples. Considering the evaluation metrics of the PLSR models, the predictive models were deemed suitable for interpreting the variations in PPO and TPC. Therefore, the next step involved visualising the prediction results to understand the browning phenomenon based on the spatial distribution of these components.

### 3.4 Visualization of distribution of PPO activity and TPC in fresh-cut potato

**Fig. 5:**
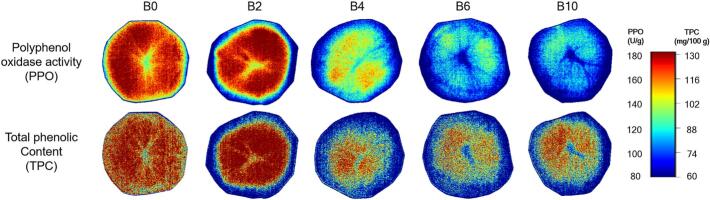
Spatial distribution maps of polyphenol oxidase (PPO) activity and total polyphenol content (TPC) in potato samples according to blanching time based on hyperspectral imaging.

**Fig. 6:**
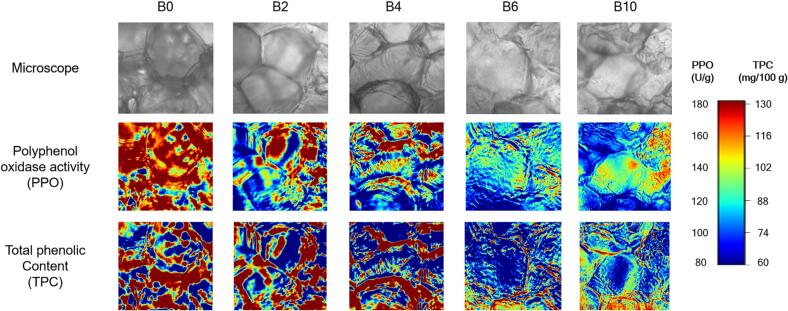
Spatial distribution maps of polyphenol oxidase (PPO) activity and total polyphenol content (TPC) in potato samples according to blanching time based on hyperspectral microscopic imaging.

HSI enables the creation of visual maps that show the distribution of quality attributes in a sample. Distribution maps were generated by integrating the spectral data from each pixel with images captured at various wavelengths and utilising prediction models (Huang et al., 2014). Notably, the browning patterns observed in the RGB images (Fig. 1) were associated with the spatial distributions of PPO activity and TPC, as shown in the hyperspectral maps (Fig. 5, Fig. 6).

Although B0 samples exhibited high levels of PPO activity and TPC distributed throughout the sample in the HSI analysis (Fig. 5), mild browning was observed across a broad area of the sample during storage (Fig. 1). This phenomenon may be attributed to the physical compartmentation of PPO and phenolic compounds. PPO is localized in plastids, such as the chloroplasts of photosynthetic cells and the leucoplasts of storage cells (Zhang, 2023), whereas phenolic compounds are primarily stored in vacuoles (Vaughn & Duke, 1984). Enzymatic browning reactions can only occur following the disruption of cellular compartmentation (Boeckx et al., 2015). As shown in Fig. 6, the HMI indicated that the B0 samples contained high levels of PPO activity and TPC within the cells, but its cellular structures were intact (Fig. 6). Therefore, the uniform and mild discoloration observed in stored B0 samples is likely attributable to the gradual interaction between PPO and TPC, which occurs over time due to the progressive breakdown of cellular compartmentation caused by oxidative stress and alterations in membrane composition and permeability during storage (Spychalla & Desborough, 1990; Wang et al., 2023).

The B2 sample displayed strong browning that originated in the vascular ring area and progressively spread outward during storage (Fig. 1). The absence of immediate browning following treatment could be attributed to the cellular structure of B2 samples. At approximately 50 °C, starch granules begin to swell as they absorb water, causing the cells to adopt a more rounded shape and facilitating cell separation (Moens et al., 2021). However, these swelling pressure exerted by gelatinised starch during blanching may have been insufficient to disrupt the connection between the middle lamella and primary cell wall, which is reinforced by the activity of pectin methyl esterase (Liu & Scanlon, 2007). Consequently, although the boundaries between cells in B2 samples became thinner than those in B0 samples, the cellular structure of B2 samples remained partially intact (Fig. 6).

However, such slight cellular damage is likely to have facilitated the disruption of compartmentation during storage, thereby promoting enhanced interactions between residual PPO and phenolic compounds. The combination of increased enzyme-substrate accessibility due to partial cell disruption and the residual PPO activity, which remained high enough to sustain rapid quinone formation in B2, likely accelerated the browning rate compared with other blanching conditions (Murata, 2022). This mechanism likely explains the rapid and pronounced browning observed in the B2 sample.

Interestingly, in the B2 sample, although PPO activity and TPC remained higher in the perimedullary zone (Fig. 5), browning did not initiate in this region but rather in the outer region (vascular ring and cortex) (Fig. 1). This phenomenon appears to be due to the restricted enzyme-substrate interaction in the structurally preserved perimedullary zone, which delayed browning. Supporting this interpretation, Fedec et al. (1977) reported that the perimedullary zone maintained higher structural integrity than the cortex after boiling in water, as a consequence of differences in cell size, the number and size of starch grains, and dry matter content. During storage, B4 samples exhibited pronounced browning that was confined to specific regions (Fig. 1). Considering the distribution maps (Fig. 5 and Fig. 6), the browning in B4 samples appears to have been driven by a mechanism similar to that described for B2 samples. However, the difference is that browning in B2 samples primarily occurred in the outer tissues, whereas in B4 samples it was expected to be restricted to localized areas where residual enzymatic activity remained.

In contrast, B6 and B10 samples did not exhibit any noticeable browning during the storage period (Fig. 1). Previous study reported that enzymatic browning is clearly evident in dead cell regions due to structural collapse that facilitates contact between PPO and phenolic compounds (Oey et al., 2017). In this study, structural collapse and cell shrinkage were indeed observed in B6 and B10 samples through HMI analysis (Fig. 6), and high TPC was also detected in the perimedullary zone (Fig. 5). However, since enzymatic browning requires the presence of both phenolic compounds and PPO, low enzyme concentrations can limit the oxidation of phenolics into quinones and their subsequent polymerisation into brown pigments (Tsikrika et al., 2022). Therefore, despite the abundance of phenolic compounds and the loss of cell compartmentation, the low PPO levels were likely suppressed the browning reaction in B6 and B10 samples.

## 4 Conclusion

This study demonstrated that hyperspectral imaging techniques (HSI and HMI), combined with PLSR, can effectively predict PPO activity and TPC levels in potatoes subjected to different blanching times. HMI showed a slightly higher accuracy than HSI, and PPO activity was predicted more reliably than TPC.

Visualization maps further revealed how blanching affected the spatial distribution of PPO, TPC, and tissue structures, thereby providing mechanistic insights into the browning phenomenon. HSI-based visualization maps enabled the identification of potential browning samples and browning regions that were difficult to distinguish by colour values immediately after blanching, by providing spatial information of PPO and TPC. From an industrial perspective, HSI offers high applicability for rapid, non-destructive monitoring without sample pretreatment.

In contrast, although HMI requires sampling of specific tissue regions for measurement, it provides visualization of cell compartmentalization and compositional information without reagent pretreatment, which cannot be obtained from HSI alone, thereby enabling the prediction of browning intensity and rate. Therefore, the integration of HSI and HMI demonstrates strong potential as an interpretative and diagnostic tool for the early detection and comprehensive understanding of browning. Nevertheless, this study was limited in terms of specific potato cultivars, the selected quality indicators (PPO and TPC), and particular wavelength ranges and algorithms, which may not fully represent the complexity of browning mechanisms under different processing conditions. In future studies, integrated HSI-HMI information could be further enhanced and explored in depth by optimizing measurement wavelengths, analytical algorithms, and fusion strategies for different potato cultivars and processing conditions according to target quality indicators.

## CRediT authorship contribution statement

**Jong-Jin Park:** Writing – original draft, Software, Methodology, Investigation, Conceptualization. **Sang Seop Kim:** Writing – original draft, Methodology, Investigation, Conceptualization. **Seul-Ki Park:** Writing – review & editing, Methodology, Investigation. **Gyuseok Lee:** Visualization, Software, Data curation. **Kee-Jai Park:** Writing – review & editing, Supervision, Resources. **Jeong-Ho Lim:** Writing – review & editing, Project administration, Conceptualization. **Jeong-Hee Choi:** Supervision, Methodology, Conceptualization. **Jeong-Seok Cho:** Writing – original draft, Supervision, Project administration, Conceptualization.

## Funding

This research was supported by the Korea Institute of Planning and Evaluation for Technology in Food, Agriculture, and Forestry (IPET) through the High Value-Added Food Technology Development Program funded by the Ministry of Agriculture, Food, and Rural Affairs (MAFRA) (Grant No. RS-2024-00410044).

## Declaration of competing interest

The authors declare that they have no known competing financial interests or personal relationships that could have appeared to influence the work reported in this paper.

## Data Availability

Data will be made available on request.
